# Age‐associated downregulation of vasohibin‐1 in vascular endothelial cells

**DOI:** 10.1111/acel.12497

**Published:** 2016-06-21

**Authors:** Eichi Takeda, Yasuhiro Suzuki, Yasufumi Sato

**Affiliations:** ^1^ Department of Vascular BiologyInstitute of Development, Aging and CancerTohoku University4‐1, Seiryo‐machi, Aoba‐kuSendai980‐8575Japan

**Keywords:** aging, angiogenesis, endothelial cell, replicative senescence

## Abstract

Vasohibin‐1 (VASH1) is an angiogenesis‐inhibiting factor synthesized by endothelial cells (ECs) and it also functions to increase stress tolerance of ECs, which function is critical for the maintenance of vascular integrity. Here, we examined whether the expression of VASH1 would be affected by aging. We passaged human umbilical vein endothelial cells (HUVECs) and observed that VASH1 was downregulated in old HUVECs. This decrease in VASH1 expression with aging was confirmed in mice. To explore the mechanism of this downregulation, we compared the expression of microRNAs between old and young HUVECs by performing microarray analysis. Among the top 20 microRNAs that were expressed at a higher level in old HUVECs, the third highest microRNA, namely miR‐22‐3p, had its binding site on the 3′ UTR of VASH1 mRNA. Experiments with microRNA mimic and anti‐miR revealed that miR‐22‐3p was involved at least in part in the downregulation of VASH1 in ECs during replicative senescence. We then clarified the significance of this defective expression of VASH1 in the vasculature. When a cuff was placed around the femoral arteries of wild‐type mice and VASH1‐null mice, neointimal formation was augmented in the VASH1‐null mice accompanied by an increase in adventitial angiogenesis, macrophage accumulation in the adventitia, and medial/neointimal proliferating cells. These results indicate that in replicative senescence, the downregulation of VASH1 expression in ECs was caused, at least in part, by the alteration of microRNA expression. Such downregulation of VASH1 might be involved in the acceleration of age‐associated vascular diseases.

## Introduction

The vascular endothelium lining the inner surface of blood vessels constitutes the interface between the blood and underlying parenchyma, and its structural as well as functional integrity is essential for the maintenance of vascular health (Schwartz, 1980). Aging of endothelial cells (ECs) alters their structure and/or function, and is a critical event resulting in deterioration of vascular integrity (Tian & Li, 2014). Indeed, vascular aging is an independent risk factor of cardiovascular diseases that impair vascular health (Lakatta & Levy, 2003) and is now recognized as a target for intervention to achieve a healthier old age (Wang & Bennett, 2012). For that reason, understanding of the significance of age‐associated changes in the vascular endothelium is essential for the development of a therapeutic strategy to maintain vascular integrity (Brunner *et al*., 2005).

The molecular mechanisms of age‐associated endothelial dysfunction are complex, but reduced bioavailability of nitric oxide (NO) has been implicated as a major player (Donato *et al*., 2015). NO maintains vascular integrity by inhibiting vasoconstriction, platelet aggregation, leukocyte adhesion, and cell proliferation through a cyclic guanosine monophosphate (cGMP)‐dependent intracellular signaling pathway, and the capacity of vascular endothelium to generate NO declines with aging (Lüscher & Barton, 1997). The vascular endothelium expresses endothelial nitric oxide synthase (eNOS) for the generation of NO. However, accumulating evidence indicates that the level of eNOS expression cannot explain the reduced availability of NO from aged ECs. Alternatively, the activity of eNOS, availability of substrate/cofactor of eNOS, and/or inactivation of generated NO are now thought to be related to the age‐associated impairment of endothelial integrity (Donato *et al*., 2015).

Angiogenesis, that is, the formation of neovessels, is one of physiological activities of ECs, and the local balance between angiogenesis stimulators and inhibitors regulates it. We recently isolated vasohibin‐1 (VASH1) as a factor synthesized by ECs that inhibits angiogenesis in an auto‐regulatory manner (Watanabe *et al*., 2004). Our recent analysis revealed that VASH1 has an additional function of increasing stress resistance of ECs by increasing the expression of superoxide dismutase 2 (SOD2) and sirtuin 1 (SIRT1; Miyashita *et al*., 2012). We therefore proposed that VASH1 provides a novel link between inhibition of angiogenesis and tolerance to vascular stress (Sato, 2015).

There are 2 types of senescence, replicative senescence and premature cell senescence. Replicative senescence represents in the narrow sense aging with telomere shortening due to replication, whereas premature cell senescence occurs acutely as the consequence of DNA damage caused by various cellular stresses (Sharpless & Sherr, 2015). Importantly, replicative senescent cells with shortened telomeres are more prone to DNA damage and resultant premature cell senescence (Oeseburg *et al*., 2010). The knockdown of VASH1 is known to cause premature senescence of ECs (Miyashita *et al*., 2012). Here, we examined whether or not replicative senescence of ECs would affect the expression of VASH1, and if so, what would be the mechanism and significance of it.

## Results

### Downregulation of VASH1 during aging

**Figure 1:**
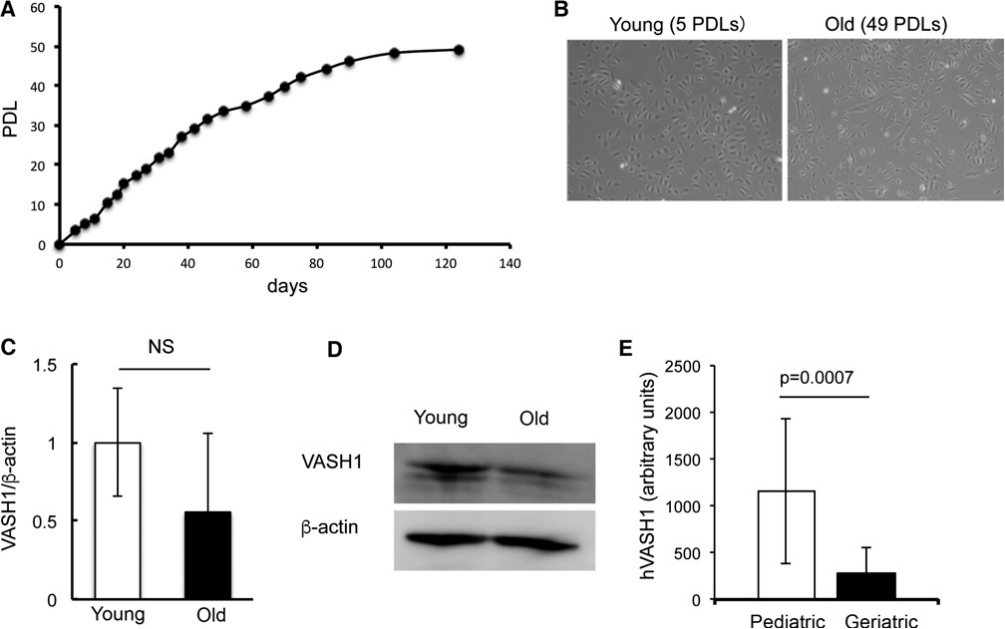
VASH1 downregulated with aging in humans. (A) Population doubling levels (PDLs) and culture days of HUVECs were plotted. (B) Morphology of young (5 PDLs) and old HUVECs (58 PDLs) is shown. (C) Young and old HUVECs were extracted, and levels of VASH1 in the extracts were determined by quantitative RT–PCR (*N *= 3). Means ± SDs are shown. The statistical significance of differences was calculated by unpaired Student's *t*‐test, and a value of *P* < 0.05 was the criterion for significance. (D) Young and old HUVECs were extracted, and levels of VASH1 in the extracts were determined by Western blotting. Experiments were repeated 3 times to confirm the reproducibility. (E) The dataset GDS2056 in the National Center for Biotechnology Information resources (http://www.ncbi.nlm.nih.gov/geo/query/acc.cgi?acc=GSE4667) was used to compare the expression of VASH1 in skeletal muscles between pediatrics (5 days to 19 years old; *N *= 3–4) and geriatrics (71–84 years old; *N *= 5). Means ± SDs are shown. The statistical significance of differences was calculated by unpaired Student's *t*‐test, and a value of *P* < 0.05 was the criterion for significance.

It is well accepted that one of the risk factors of vascular diseases is endothelial cell senescence (Tian & Li, 2014). Here, we tested whether or not endothelial cell senescence would affect the expression of VASH1. We serially passaged HUVECs to cause replicative senescence *in vitro* (Fig. 1A). The morphology of young and old HUVECs is shown in Fig. 1B. The expression of p16 was increased, whereas that of SIRT1 was decreased in old HUVECs (Fig. S1). We then compared the expression of VASH1. VASH1 mRNA tended to decrease (Fig. 1C) and VASH1 protein apparently decreased in the old HUVECs (Fig. 1D). To confirm this change in VASH1 expression during aging *in vivo*, we applied the dataset GDS2056 (Kang *et al*., 2005), which is a microarray dataset that was prepared to compare the expression profiles in skeletal muscles between pediatrics (5 days to 19 years old) and geriatrics (71–84 years old). By using this dataset, we could confirm the significant decrease in VASH1 expression during aging in humans (Fig. 1E).

**Figure 2:**
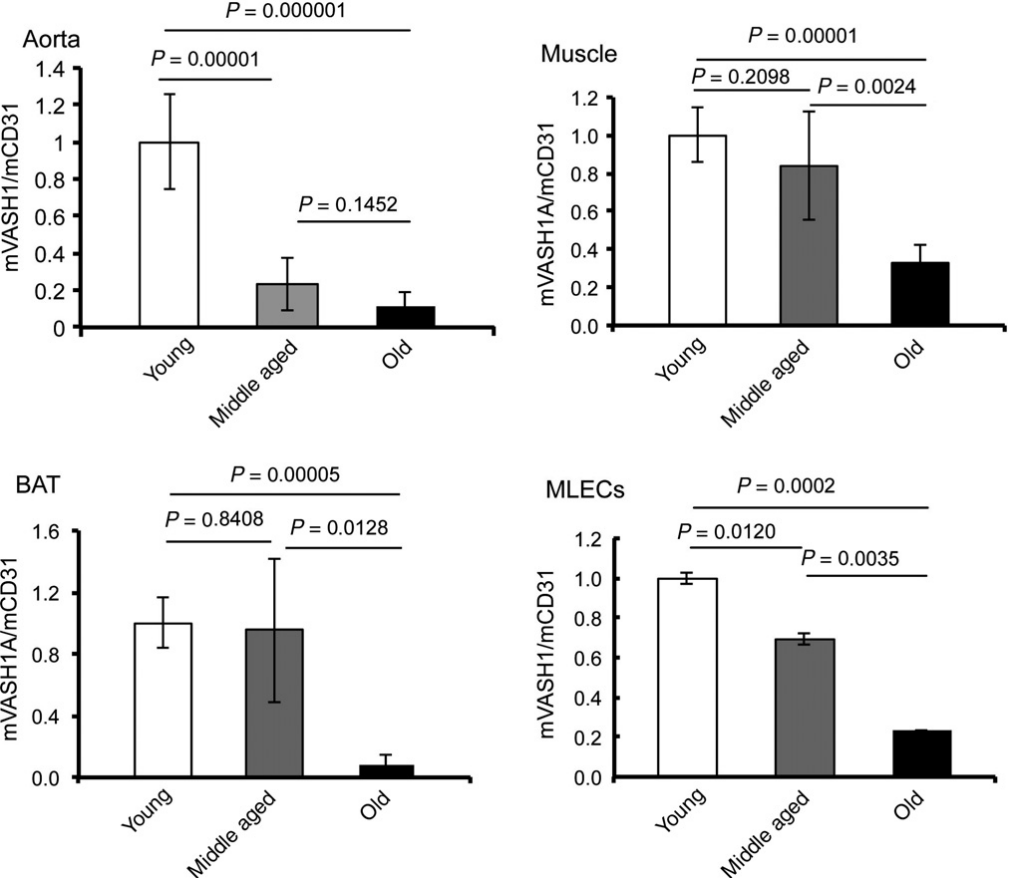
VASH1 downregulated with aging in mice. Samples were obtained from thoracic aorta, brown adipose tissue (BAT), skeletal muscles, or isolated murine lung ECs (MLECs) of young (8 weeks old, *N *= 8), middle‐aged (52–55 weeks old, *N *= 7–8), and old (2 years old, *N *= 4–6) mice, and the expression of VASH1 mRNA was compared. Means ± SDs are shown. The statistical significance of differences was calculated by one‐way ANOVA followed by Tukey post hoc test, and a value of *P* < 0.05 was the criterion for significance.

Changes in the expression of VASH1 during aging were further examined in mice. We obtained samples from young (8 weeks old), middle‐aged (52–55 weeks old), and old (2 years old) mice and compared the expression of VASH1 in selected tissues and isolated ECs. The expression of VASH1 was decreased in specimens from the old mice (Fig. 2). Importantly, the decrease in VASH1 expression was evident earlier and most pronounced in the aorta (Fig. 2).

### Alteration of microRNA expression may cause the downregulation of VASH1 during aging

**Figure 3:**
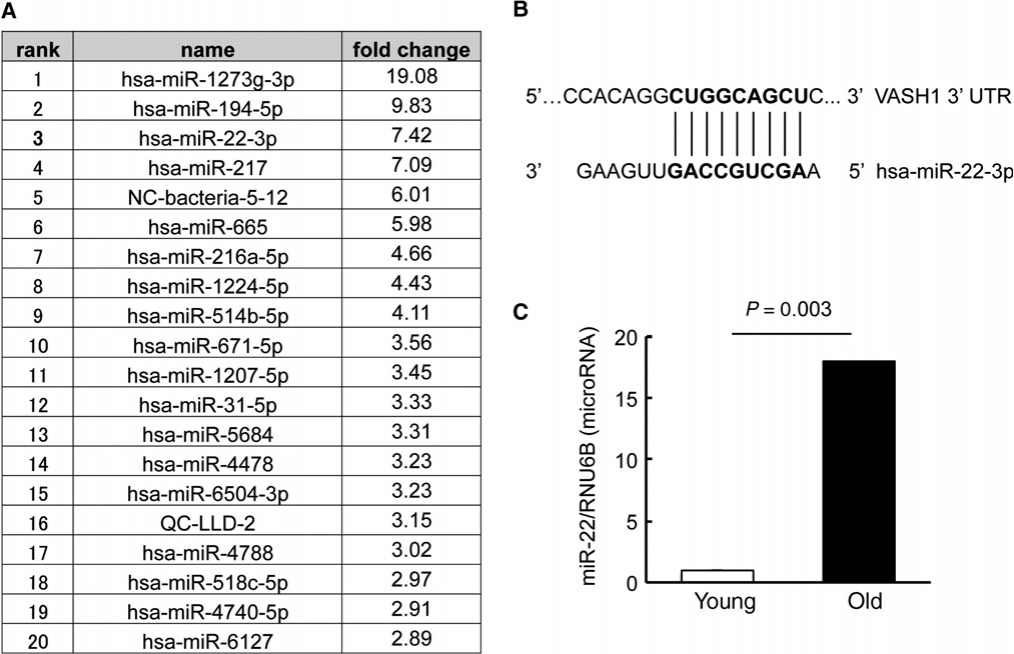
A candidate of a senescence‐associated microRNA in HUVECs that could modulate the expression of VASH1. (A) Samples were obtained from young and old HUVECs, and the expression profile of microRNAs was compared by the microarray method. The top 20 microRNAs and their fold increases are shown. (B) Only miR‐22‐3p had its binding site on the 3′ UTR of VASH1 mRNA. The sequence of miR‐22‐3p and its binding site on the 3′ UTR of hVASH1 are shown. (C) The expression of miR‐22‐3p in young or old HUVECs was determined by qRT–PCR and compared (*N *= 3). Means ± SDs are given. The statistical significance of differences was calculated by unpaired Student's *t*‐test, and a value of *P* < 0.05 was the criterion for significance.

To find the cause of this decrease in VASH1 expression, we focused our attention on microRNAs. We obtained samples from young and old HUVECs and compared the expression profile of microRNA by performing microarray analysis. We picked up the top 20 microRNAs with augmented expression in the old HUVECs (Fig. 3A). We then screened these microRNAs for those having a binding site on the 3′ UTR of VASH1 mRNA and found only 1 such microRNA, namely miR‐22‐3p (Fig. 3B). The increased expression of miR‐22‐3p in the old HUVECs was further confirmed by the results of quantitative PCR (Fig. 3C).

**Figure 4:**
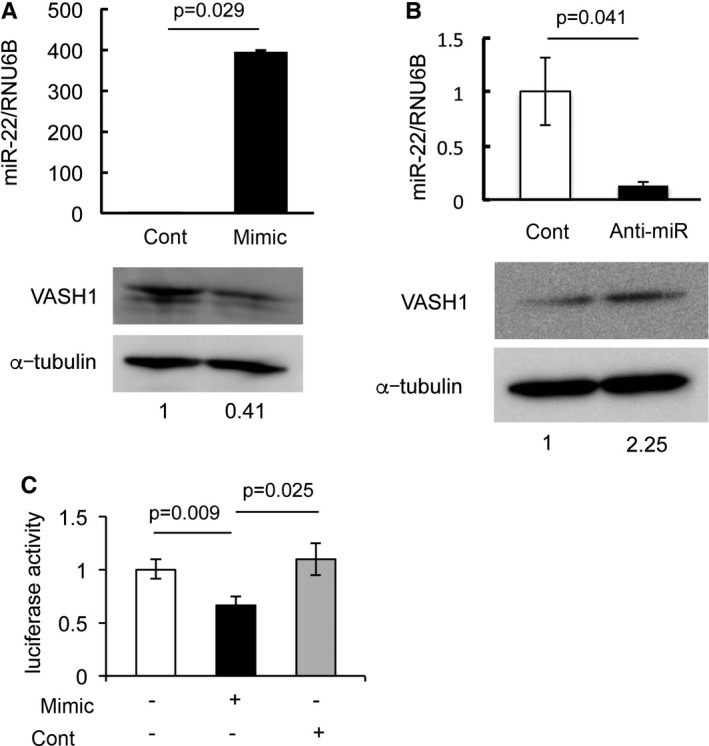
miR‐22‐modulated expression of VASH1 in HUVECs. (A) Young HUVECs (3–5 PDLs) were transfected with miR‐22 mimic, and their VASH1 level was compared with that of the control. Experiments were repeated 3 times to confirm the reproducibility. (B) Old HUVECs (42–45 PDLs) were transfected with a miR‐22 inhibitor, and their VASH1 level was compared with that of the control. Experiments were repeated 3 times to confirm the reproducibility. (C) Luciferase assay was performed as described in Materials and Methods. Means ± SDs are given (*N *= 3). The statistical significance of differences was calculated by unpaired Student's *t*‐test for A and B, or by one‐way ANOVA followed by Tukey post hoc test for C, and a value of *P* < 0.05 was the criterion for significance.

We then tested whether miR‐22‐3p would affect the expression of VASH1 in HUVECs. When a miR‐22‐3p mimic was used for transfection of young HUVECs, the expression of VASH1 was decreased (Fig. 4A). Alternatively, when old HUVECs were transfected with anti‐miR‐22‐3p, the expression of VASH1 was increased (Fig. 4B). Moreover, a miR‐22‐3p mimic suppressed the luciferase activity of HEK293 cells transfected with pmiRGLO‐hVASH1‐3′ UTR vector (Fig. 4C). These results indicate that the increase in miR‐22‐3p may have played a role, at least in part, in the downregulation of VASH1 expression during aging.

### Role of VASH1 in the development of neointimal formation and atherosclerotic lesion

**Figure 5:**
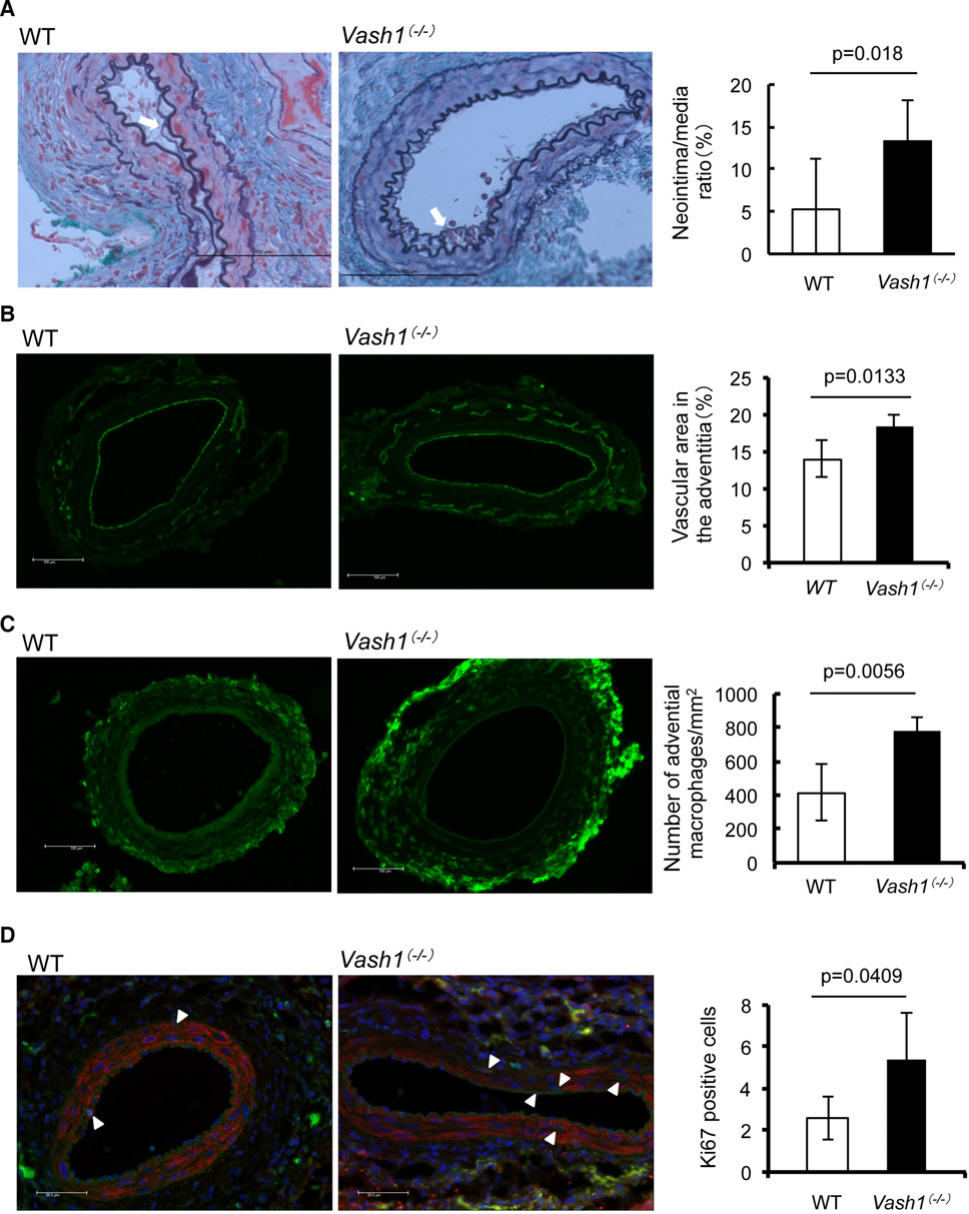
Comparison of neointimal formation between WT and *Vash1* ^*(−/−)*^ mice in the cuff injury model. The cuff injury model was applied to WT and *Vash1* ^*(−/−)*^ mice, and neointimal formation was compared as described in Materials and Methods. (A) Elastica‐Masson staining was performed. White arrows indicate the neointima. Bar = 100 μm. Neointimal formation was quantified and compared between WT and *Vash1* ^*(−/−)*^ mice (*N *= 7). (B) Immunostaining of CD31 was performed. Bar = 100 μm. The vascular area in the adventitia was quantified and compared between WT and *Vash1* ^*(−/−)*^ mice (*N *= 5). (C) Immunostaining of F4/80 was performed. Bar = 100 μm. The number of adventitial macrophages was quantified and compared between WT and *Vash1* ^*(−/−)*^ mice (*N *= 5). (D) Immunostaining of Ki67 (green) and alpha SMA (red) was performed. Nuclei were visualized by DAPI (blue). Bar = 100 μm. Arrowheads indicate Ki67‐ and alpha SMA‐positive cells in the media and neointima. Those cells were quantified and their number compared between WT and *Vash1* ^*(−/−)*^ mice (*N *= 6). Bar = 100 μm. Means ± SDs are given. The statistical significance of differences was calculated by unpaired Student's *t*‐test, and a value of *P* < 0.05 was the criterion for significance.

The decrease in VASH1 expression in the aged aorta, as was shown in Fig. 2, could be a risk factor of vascular diseases. We wanted to test the significance of this decrease in VASH1. To do so, we used *Vash1* ^*(−/−)*^ mice and applied a cuff injury to the femoral artery. As shown in Fig. 5A, neointimal formation was significantly augmented in the *Vash1* ^*(−/−)*^ mice. Neovessels in the adventitia were significantly enlarged, and more macrophages accumulated in the adventitia of the *Vash1* ^*(−/−)*^ mice (Fig. 5B and C) compared with those in the wild‐type. Proliferating cells in intima and media of the injured artery were significantly increased in number in the *Vash1* ^*(−/−)*^ mice (Fig. 5D). These results indicate that the loss of endogenous VASH1 was responsible for the advanced neointimal formation.

Next, we examined the expression of various molecules in the cuff‐injured femoral arteries (Fig. S2). Although we did not see any significant differences, TNF‐α expression tended to be higher and CD206 to be lower in the *Vash1* ^*(−/−)*^ mice. We thus speculated that the accumulated macrophages in the *Vash1* ^*(−/−)*^ mice were rather polarized to M1. Also, VEGF expression was lower in the *Vash1* ^*(−/−)*^ mice, which might have been due to the adaptation to the lack of the endogenous angiogenesis inhibitor VASH1. MCP‐1, ICAM‐1, and VCAM‐1 levels were unchanged. We therefore speculate that the increased neovascularization in the adventitia *per se* might have been responsible for this accumulation of macrophages.

**Figure 6:**
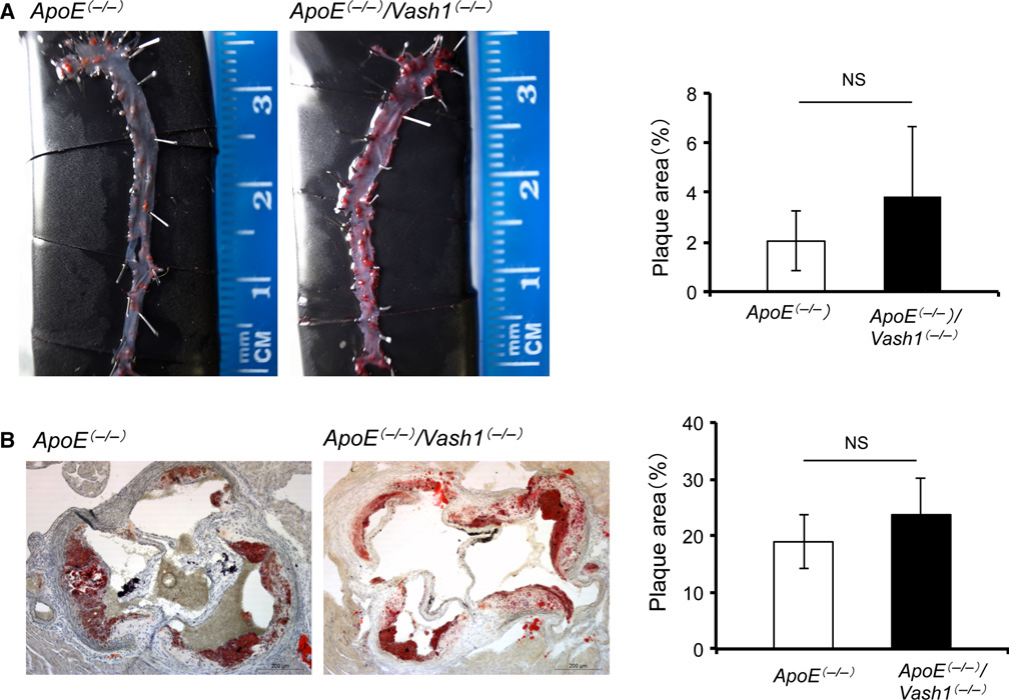
Comparison of atherosclerotic lesions between *ApoE* ^*(−/−)*^ and *ApoE* ^*(−/−)*^ */Vash1* ^*(−/−)*^ mice. *ApoE* ^*(−/−)*^ and *ApoE* ^*(−/−)*^ */Vash1* ^*(−/−)*^ mice were fed a HFD for 12 weeks, and atherosclerotic lesions were compared as described in Materials and Methods. (A) The result of Oil Red O staining of atherosclerotic lesions in whole aorta *en face* is shown. The plaque area was determined as the Oil Red O‐positive area/total descending aorta area (%). (B) Oil Red O staining of atherosclerotic lesions in the aortic valves is shown. The plaque area was determined as the Oil Red O‐positive area/total aortic valve area (%). *N *= 8, Bar = 200 μm. Means ± SDs are given. The statistical significance of differences was calculated by unpaired Student's *t*‐test, and a value of *P* < 0.05 was the criterion for significance.

We next applied *ApoE* ^*(−/−)*^ mice to examine the development of atherosclerotic lesions. We obtained *ApoE* ^*(−/−)*^ */Vash1* ^*(−/−)*^ mice by crossing *ApoE* ^*(−/−)*^ mice and *Vash1* ^*(−/−)*^ mice, fed them a high fat diet (HFD), and examined the extent of atherosclerotic lesions. Although we did not see a significant difference, lipid accumulation tended to be more advanced and to have spread around the entire circumference in the *ApoE* ^*(−/−)*^ */Vash1* ^*(−/−)*^ mice (Fig. 6). When various serum parameters were compared between *ApoE* ^*(−/−)*^ mice and *ApoE* ^*(−/−)*^ */Vash1* ^*(−/−)*^ mice, only HCL‐C was elevated in the *ApoE* ^*(−/−)*^ */Vash1* ^*(−/−)*^ mice (Fig. S3).

## Discussion

It is well accepted that one of the risk factors of vascular diseases is aging, as the prevalence of cardiovascular diseases is significantly increased in the older population (Hazzard, 1989). Aging is associated with endothelial cell dysfunction, and endothelial cell dysfunction is thought to be critical for the development of various vascular diseases (Wang & Bennett, 2012). However, the entire picture of age‐related endothelial dysfunction still remains to be clarified. Earlier we found that VASH1 plays a critical role in the stress resistance of ECs by regulating the expression of SOD2 and SIRT1 in ECs (Miyashita *et al*., 2012). Here, we showed that the expression of VASH1 was significantly decreased during replicative senescence of ECs. Thus, we propose this decrease in VASH1 expression to be one of the causes of age‐related endothelial dysfunction and may relate to age‐associated vascular diseases.

We further extended our analysis to clarify how replicative senescence affected the expression of VASH1 in ECs and focused our attention on microRNAs. MicroRNAs are small ~22‐nucleotide noncoding RNAs that are present in both plants and animals. It is estimated that approximately one‐third of all animal genes are controlled by microRNAs, and thus, microRNAs exhibit a wide range of biological processes including aging (Breving & Esquela‐Kerscher, 2010). Indeed, several earlier studies were directed to examine the relationship between microRNAs and endothelial cell senescence (Menghini *et al*., 2009; Zhao *et al*., 2010; Jong *et al*., 2013; Olivieri *et al*., 2013; Zhu *et al*., 2013). Our present comprehensive microarray analysis revealed that the microRNA with the third highest augmented expression in senescent HUVECs was miR‐22‐3p and that it had a binding site on the 3′ UTR of VASH1 mRNA. Our subsequent analyses using microRNA mimic and anti‐miR showed that miR‐22‐3p might be involved, at least in part, in the downregulation of VASH1 in ECs during replicative senescence.

MiR‐22 was initially defined as one of the senescence‐associated microRNAs that represses cancer progression by inducing cell senescence (Xu *et al*., 2011). However, a subsequent analysis revealed that miR‐22 is most prominently upregulated during cardiovascular aging, targeting minecan (osteoglycin, OGN; Jazbutyte *et al*., 2013). More recently, miR‐22 was found to promote senescence of endothelial progenitor cells by targeting AKT3 (Zheng & Xu, 2014). Here, we added VASH1 to the list of miR‐22 targets that are downregulated during endothelial cell senescence and that might be response for aging‐associated vascular diseases including atherosclerosis.

The decrease in VASH1 expression during aging was evident earlier and most pronounced in the aorta (Fig. 2). We therefore examined the role of VASH1 in the age‐related arterial disease, namely arteriosclerosis. We first applied the femoral artery cuff injury model to examined neointimal formation of artery. When the cuff injury model was performed, there was a significant increase in neointimal formation in the *Vash1* ^*(−/−)*^ mice. The area of adventitial vessels was clearly enlarged, and macrophage accumulation in the adventitia was significantly augmented in the *Vash1* ^*(−/−)*^ mice. We previously demonstrated that exogenous VASH1 prevents neointimal formation together with neovascularization and macrophage accumulation in the adventitia (Yamashita *et al*., 2006). VASH1 is an endothelium‐derived factor with dual functions: One is inhibition of angiogenesis, and the other is promotion of stress resistance (Watanabe *et al*., 2004; Miyashita *et al*., 2012). We therefore speculate that the decreased stress resistance of ECs in *Vash1* ^*(−/−)*^ mice was rather responsible for this increased macrophage accumulation in the adventitia and that it promoted the neointimal formation.

We then advanced our analysis to examine the development of atherosclerosis by crossing *Vash1* ^*(−/−)*^ and *ApoE* ^*(−/−)*^ mice. Our analysis revealed that, although it was not statistically significant, the atherosclerotic region tended to be advanced in the *ApoE* ^*(−/−)*^/*Vash1* ^*(−/−)*^ mice. We recently reported that diabetic vascular complications are more advanced in *Vash1* ^*(−/−)*^ mice (Hinamoto *et al*., 2014). Our present study along with the recent one just mentioned indicates that endogenous VASH1 plays a crucial role in the prevention of vascular diseases.

In summary, our analysis revealed for the first time that VASH1 was downregulated during replicative senescence of ECs. This senescence‐mediated downregulation of VASH1 in ECs might be caused by the alteration (upregulation) of microRNA expression, namely that of miR‐22. Such downregulation of VASH1 might be involved in the acceleration of age‐associated vascular diseases. The aging mouse model is currently underway to examine the significance of senescence‐mediated downregulation of VASH1 in ECs.

## Experimental procedures

### Cell culture

Human umbilical vein endothelial cells (HUVECs) were purchased from Lonza (Basel, Switzerland) and cultured on type I collagen‐coated dishes (Iwaki, Chiba, Japan) in EBM2 (Lonza) containing endothelial cell growth supplements and 2% fetal bovine serum. HUVECs were harvested by using 0.025% trypsin/EDTA solution (Sigma‐Aldrich, St Louis, MO, USA) and passaged when they were confluent. Population doubling (PD) was calculated according to the method described previously (Maciag *et al*., 1981). Briefly, the number of PDs was calculated by using the following formula: PD = log2 (Ch/Cs), where Ch is the viable cells number at harvest and Cs is the number of cells seeded. Population doubling levels (PDLs) were calculated as the sum of all PD changes. We defined young HUVECs as cells with <10 PDLs and old HUVECs as those with more than 40 PDLs.

### Transfection

HUVECs (2 × 10^5^ cells) were transfected with mirVana hsa‐miR‐22‐3p mimic, miRNA mimic negative control #1, hsa‐miR‐22‐3p inhibitor, or miRNA inhibitor negative control #1 (Applied Biosystems, Foster City, CA, USA) in Lipofectamine RNAiMAX (Invitrogen, Carlsbad, CA, USA) containing Opti‐MEM I (Thermo Fisher Scientific Inc., Rockford, IL, USA), each at a final concentration of 30 nmol L^−1^. The cells were then incubated for 48 or 72 h prior to the experiments.

### Luciferase assay

The pmirGLO‐hVASH1‐3′ UTR was constructed from pmirGLO Dual‐Luciferase miRNA Target Expression Vector (Promega, Madison, WI, USA) with entire human VASH1 3′ UTR sequences. Human embryonic kidney (HEK293) cells were transfected with this vector in the presence or absence of hsa‐miR‐22‐3p mimic or mimic negative control #1. Thereafter, HEK293 cells were cultured for 24 h and assayed with the Dual‐Luciferase Reporter Assay System (Promega). Luciferase activities were calculated by firefly luciferase activity/Renilla luciferase activity.

### Western blot analysis

Western blot analysis was performed as described previously (Miyashita *et al*., 2012). Briefly, cells were lysed with RIPA buffer (Nacalai Tesque, Shiga, Japan), and the cell lysates were then subjected to the polyacrylamide gel electrophoresis. Next, the separated proteins were transferred to the membranes, which were blocked for 1 h at room temperature with Tris–HCl‐buffered saline (TaKaRa Bio, Shiga, Japan) containing 0.05% Tween‐20 (T‐TBS) and 2.5% skim milk. After the transfer, the membranes were incubated for 1 h at room temperature with HRP‐conjugated anti‐human VASH1 mAb (4E12, 1:1000 dilution; Watanabe *et al*., 2004), anti‐human β‐actin monoclonal antibody (1:10000; Sigma‐Aldrich), or anti‐human α‐tubulin monoclonal antibody (1:1000 dilution; Merck Millipore, Billerica, MA, USA). After the membranes had been washed 3 times with T‐TBS, they were incubated for 1 h at room temperature with anti‐rat IgG (whole molecule)‐peroxidase antibody or anti‐mouse IgG (whole molecule)‐peroxidase antibody (Sigma‐Aldrich) as a secondary antibody. They were then washed again 3 times with T‐TBS, after which the blots were detected by an enhanced chemiluminescence method using an Immobilon Western HRP Substrate (Merck Millipore). The results were visualized by using an LAS‐4000 (Fuji Film, Tokyo, Japan).

### Microarray analysis of microRNAs

MicroRNAs (miRNAs) were isolated from HUVECs with a miRNeasy mini kit (QIAGEN, Tokyo, Japan). Samples were outsourced to Filgen Array miRNA Homo sapiens (Filgen, Nagoya, Japan) and used to compare miRNA expression in young and old HUVECs.

### Quantitative reverse transcription real‐time polymerase chain reaction (qRT–PCR)

For the quantification of miRNAs, real‐time qRT–PCR was performed with a TaqMan miRNA reverse transcription kit (Applied Biosystems) used according to the manufacturer's protocol. Small nucleolar RNA (RNU6B) was used as the housekeeping small RNA reference gene.

For the quantification of mRNAs, qRT–PCR performed as described previously (Suenaga *et al*., 2014). Briefly, total RNA was prepared from MLECs by using an RNeasy mini kit (QIAGEN) and from mice tissues by using ISOGEN II (Nippon Gene, Tokyo, Japan) according to the manufacturer's instructions. Single‐stranded complementary DNA (cDNA) was synthesized by using ReverTra Ace (TOYOBO, Tokyo, Japan). PCR was performed with a thermal cycler system (CFX‐96 Real‐Time system, C1000 Thermal Cycler; Bio‐Rad, Hercules, CA, USA) and SYBR Premix Ex Taq (TaKaRa Bio). β‐actin or CD31 was used as the reference gene. The primer pairs are shown in Table S1.

### Animal studies

All of the animal studies were approved by the Center for Laboratory Animal Research of Tohoku University. Male wild‐type (WT) and *Vash1* ^*(−/−)*^ mice on a C57BL/6J background (Kimura *et al*., 2009) were mated and maintained with normal chow (CE‐2; CLEA Japan Inc., Tokyo, Japan). We defined C57BL/6J mice at the age of 8 weeks as young, of 52–55 weeks as middle aged, and of 24–26 months as old (Honek *et al*., 2014). To establishment *ApoE* ^*(−/−)*^/*Vash1* ^*(−/−)*^ mice, we mated *Vash1* ^*(−/−)*^ mice and *ApoE* ^*(−/−)*^ (Zhang *et al*., 1992) mice (The Jackson Laboratory, Bar Harbor, Me, USA) to obtain heterozygous *ApoE* ^*(+/−)*^/*Vash1* ^*(+/−)*^ mice. We then intercrossed those double heterozygotes mice to obtain homozygous *ApoE* ^*(−/−)*^/*Vash1* ^*(−/−)*^ mice. Genotyping was shown in Fig. S4.

#### Isolation of Mouse Lung Endothelial Cells (MLECs) and excision of certain mouse tissues

Isolation of MLECs was performed as described previously (Ito *et al*., 2013). Briefly, MLECs were isolated from mouse lung tissue by the use of a Magnetic Cell Sorting System device (MACS; Miltenyi Biotec, Auburn, CA, USA). Blood cells were removed from lungs by PBS perfusion before excision. The lungs were minced and digested with collagenase D (Roche Diagnostics, Indianapolis, IN, USA), after which the cell suspensions were filtered through a 70‐μm cell strainer (BD Falcon; BD Biosciences, San Jose, CA, USA). The filtered cells were then treated with red blood cell lysing buffer (8.3 g L^−1^ NH_4_Cl in 0.01 mol L^−1^ Tris–HCl), and CD31‐positive MLECs were isolated by MACS with microbeads conjugated with monoclonal anti‐mouse CD31 antibodies. Selected tissues were excised from the sacrificed mice, snap‐frozen, and stored at −80°C prior to examination.

#### Femoral artery cuff injury model

The femoral artery cuff injury model was prepared as described previously (Moroi *et al*., 1998; Yamashita *et al*., 2006; Schwaiberger *et al*., 2010; Saito *et al*., 2013). Briefly, one of the femoral arteries of 8‐ to 9‐week‐old male WT or *Vash1(−/−)* mice was isolated from the surrounding tissues and then sheathed with a 2.0‐mm polyethylene cuff made of PE‐50 tubing (inner diameter, 0.56 mm; outer diameter, 0.965 mm; Becton Dickinson, Franklin Lakes, NJ, USA). The contralateral femoral artery was dissected from the surrounding tissues without cuff placement (sham‐operated). For analysis by qRT–PCR, 1 week after the cuff placement, the femoral arteries were excised, snap‐frozen, and stored at −80°C until they were used for qRT–PCR. For histological analysis 2 weeks after the cuff placement, the femoral arteries were excised; fixed in 10% neutral buffered formalin (Wako Chemicals, Osaka, Japan); kept overnight at 4°C in 10, 20, 30% sucrose in PBS; and then embedded in O.C.T compound (Sakura Finetek, Tokyo, Japan). Thereafter, they were snap‐frozen and stored at −80°C until further processed. The arteries were cut into 10 serial cross‐sections (5 μm) with an interval of 100 μm between sections and subsequently stained with Elastica‐Masson (EM). The areas of intima and media were measured by using ImageJ, and the ratio of the intimal area to the medial area (I/M ratio) was calculated. In 10 similar sections, the area of CD31‐positive vessels per adventitial area and the area of F4/80‐positive macrophages per adventitial area were counted and normalized to 1 mm^2^. Also, the number of Ki67‐positive cells per intimal and medial area was counted.

#### Immunofluorescence analysis of femoral arteries

Cryosections were washed in PBS and then blocked for 1 h at room temperature in blocking solution (PBS containing 5% normal serum and 0.1% Tween‐20). After removal of the blocking solution, the sections were reacted overnight at 4°C with rat anti‐mouse CD31 antibody (1:400 dilution; BD Biosciences), rat anti‐mouse F4/80 antibody (1:100 dilution; AbD Serotec, Raleigh, NC, USA), mouse anti‐α‐smooth muscle antibody (1:100 dilution; Sigma‐Aldrich), or rabbit anti‐Ki67 antibody (1:100 dilution; Thermo Fisher Scientific Inc.) 4°C followed by Alexa 488‐ or Alexa 594‐conjugated secondary antibodies (1:2000 dilution; Jackson ImmunoResearch Laboratories, West Grove, PA, USA) and DAPI for 1 h at room temperature. Finally, the specimens were mounted with fluorescent mounting medium (DAKO Corporation, Carpinteria, CA, USA) and images were acquired by using a fluorescence microscope (DMI6000B; Leica Microsystems, Wetzlar, Germany).

#### Diet‐induced atherosclerosis

Diet‐induced atherosclerosis in mice was performed as described previously (Chiba *et al*., 2008; Usui *et al*., 2012; García *et al*., 2013). Briefly, male *ApoE* ^*(−/−)*^ and *ApoE* ^*(−/−)*^/*Vash1* ^*(−/−)*^ mice were fed a HFD (D12079B, Research Diets, Inc., New Brunswick, NJ, USA) for 12 weeks starting from 8 weeks of age.

#### Blood sample analysis

For blood sampling, mice were sacrificed after a 16‐h fast; and blood samples were taken by cardiopuncture. The serum prepared by centrifugation (2000 g, 20 min, 4°C). For measuring certain parameters, samples were outsourced to ORIENTAL YEAST CO., LTD (Tokyo, Japan) for serum analysis.

#### Analysis of atherosclerotic lesions

Aortas were obtained and evaluated as Oil Red O‐stained areas, as described previously (Ishigaki *et al*., 2008). Briefly, the aorta was dissected from surrounding tissues, cut open with the luminal surface facing up, and then immersion‐fixed in 10% neutral buffered formalin. Thereafter, the inner aortic surfaces were stained with Oil Red O (Sigma‐Aldrich) for 30 min at room temperature. For aortic root cross‐section analysis, the aortic root and ascending aorta were excised from mice, rinsed in PBS, frozen in O.C.T. compound, sectioned into 8‐μm thick serial sections at intervals of 100 μm by using a cryostat. These sections were stained with Oil Red O and counterstained with hematoxylin. Images of atherosclerotic plaque areas were captured by using a microscope (DM2000 LED, Leica Microsystems), and the average lesion area was determined by measuring 6 sections from each mouse by ImageJ.

### Statistical analysis

Data were expressed as means ± SD. The statistical significance of differences between groups and *P* values was calculated by using unpaired Student's *t*‐test or by one‐way ANOVA followed by Tukey post hoc test. A value of *P *< 0.05 was the criterion for significance.

## Funding

A grant from the Global COE for Conquest of Signal Transduction Diseases with Network Medicine, Tohoku University.

## Conflict of interest

The authors declared that they have no conflict of interest.

## Author contributions

The experiments were designed by SaY. All the experiments were performed by ET, and animal studies were supervised by SuY. The manuscript was prepared by ET and SaY.

## Supporting information


**Fig. S1** Expression of p16 and SIRT1.

**Fig. S2** Expression of various genes in the femoral arteries that might be related to neointimal formation.

**Fig. S3** Biochemical analysis of sera *ApoE^*(−/−)*^* and *ApoE^*(−/−)*^*/Vash1^*(−/−)*^ mice. Total cholesterol (T‐CHO), high‐density lipoprotein cholesterol (HDL‐C), triglyceride (TG), total lipid (TL), non‐esterified fatty acid (NEFA), phospholipid (PL), aspartate aminotransferase (AST), alanine aminotransferase (ALT), lactate dehydrogenase (LDH), choline esterase (ChE), and blood glucose (Glu) were determined and compared.

**Fig. S4** Genotyping of mice.

**Table S1** Primer list.
