# Supplementary figures and images for: Age‐associated downregulation of vasohibin‐1 in vascular endothelial cells
Source: Aging Cell. 2016 Jun 21;15(5):885–92. doi: 10.1111/acel.12497 (PMC5013028; doi:10.1111/acel.12497)

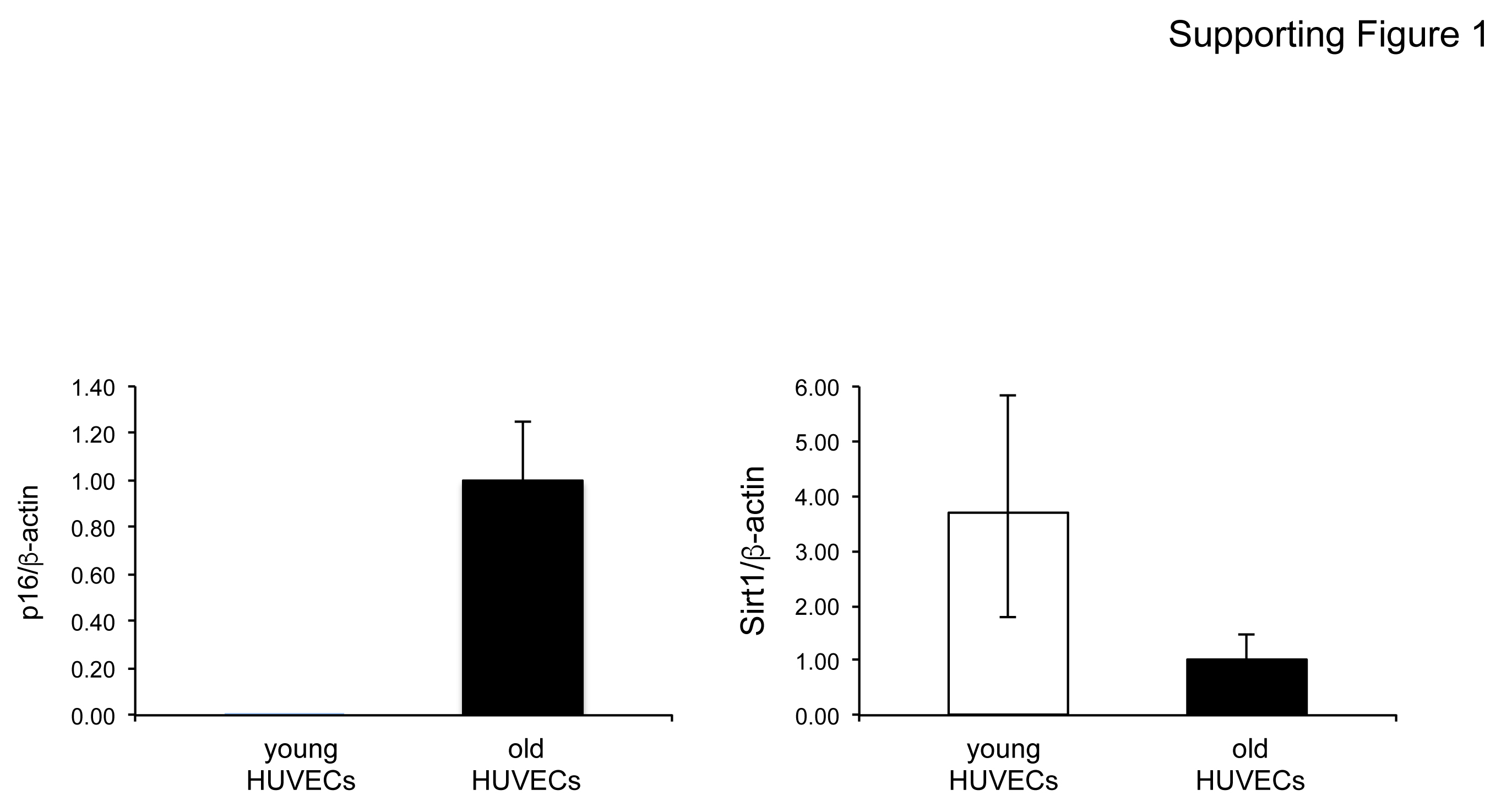

Supplement: Supplementary file 1 — Fig. S1 Expression of p16 and SIRT1. [file ACEL-15-885-s001.jpg]

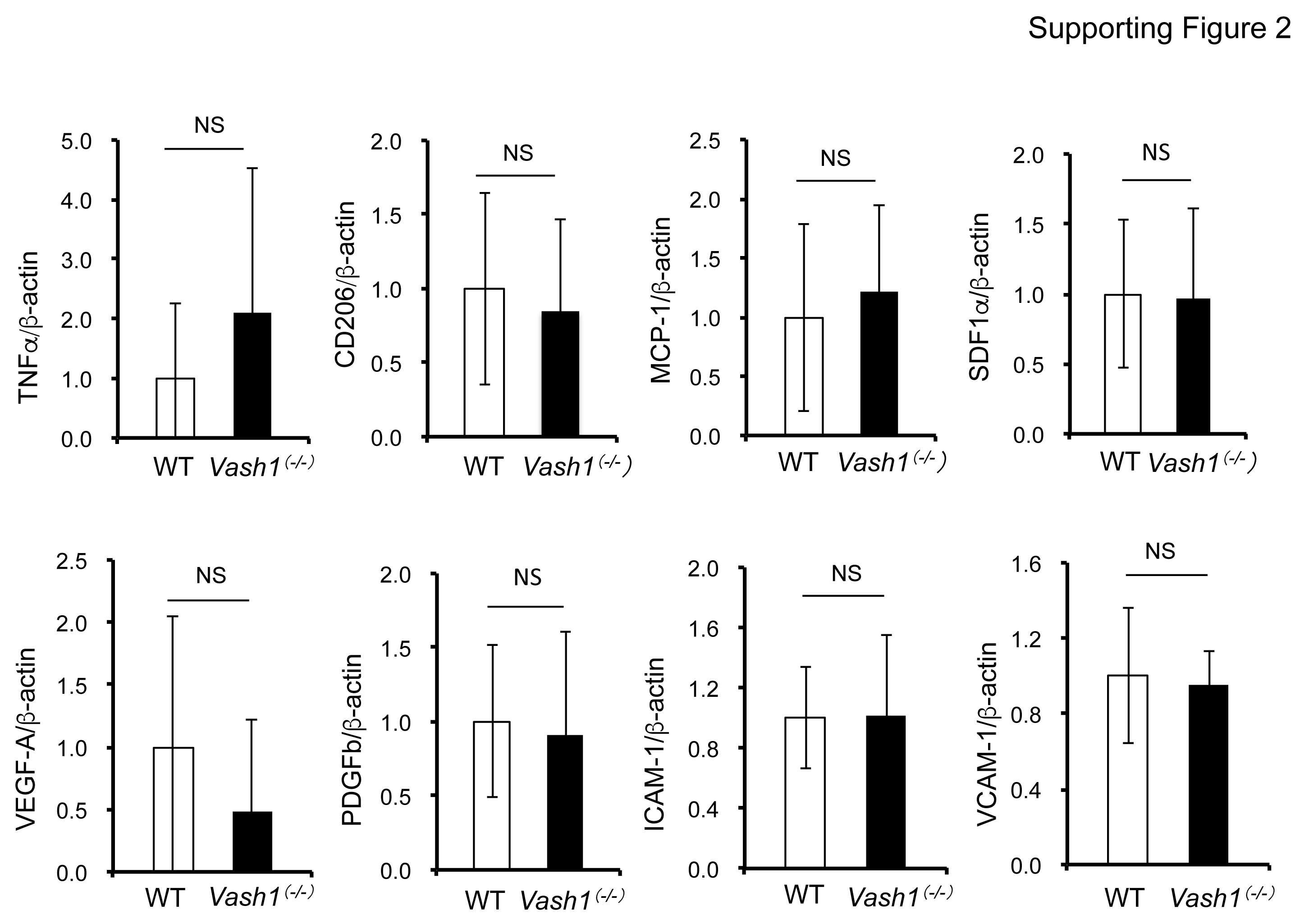

Supplement: Supplementary file 2 — Fig. S2 Expression of various genes in the femoral arteries that might be related to neointimal formation. [file ACEL-15-885-s002.jpg]

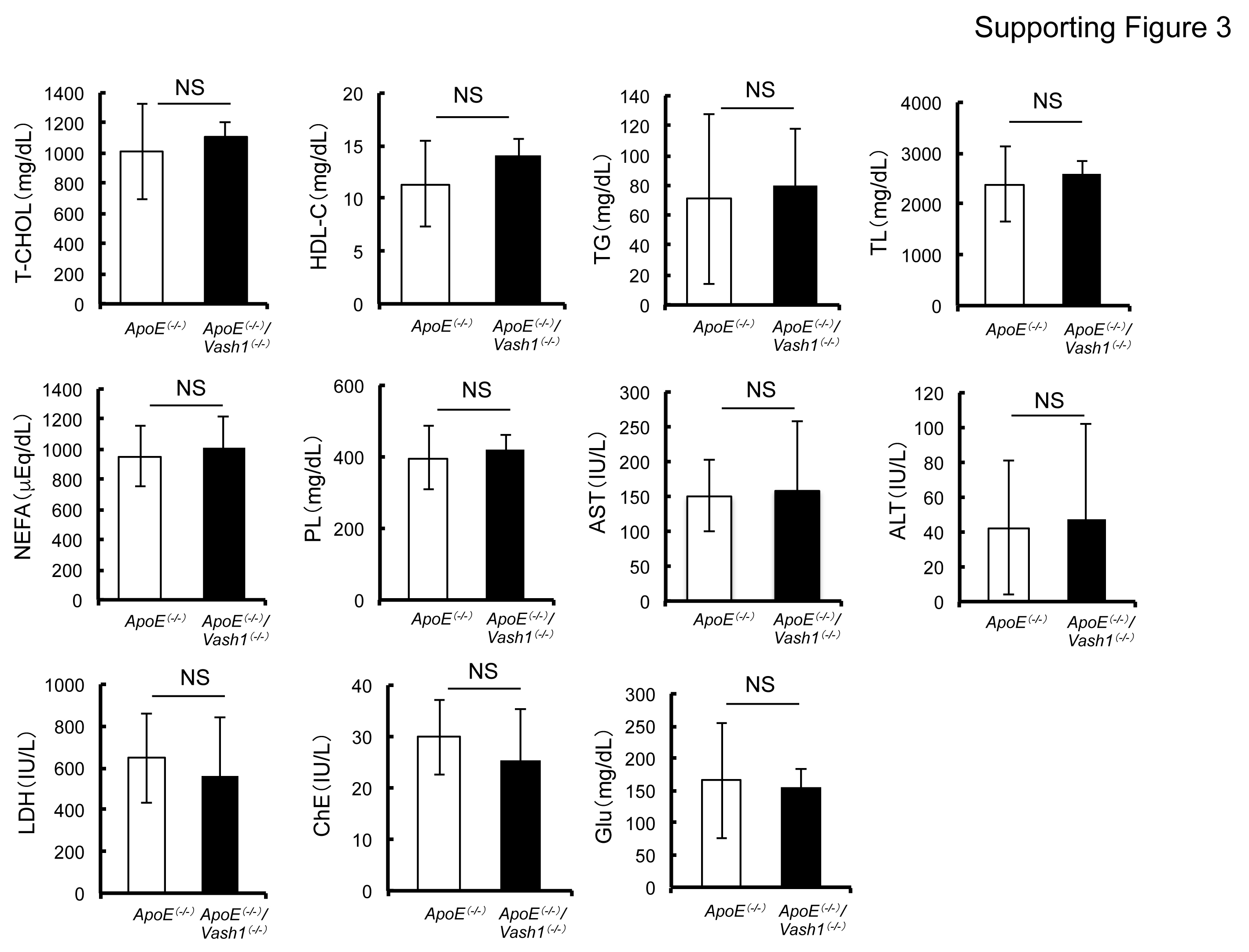

Supplement: Supplementary file 3 — Fig. S3 Biochemical analysis of sera ApoE(−/−) and ApoE(−/−)/Vash1(−/−) mice. Total cholesterol (T‐CHO), high‐density lipoprotein cholesterol (HDL‐C), triglyceride (TG), total lipid (TL), non‐esterified fatty acid (NEFA), phospholipid (PL), aspartate aminotransferase (AST), alanine aminotransferase (ALT), lactate dehydrogenase (LDH), choline esterase (ChE), and blood glucose (Glu) were determined and compared. [file ACEL-15-885-s003.jpg]

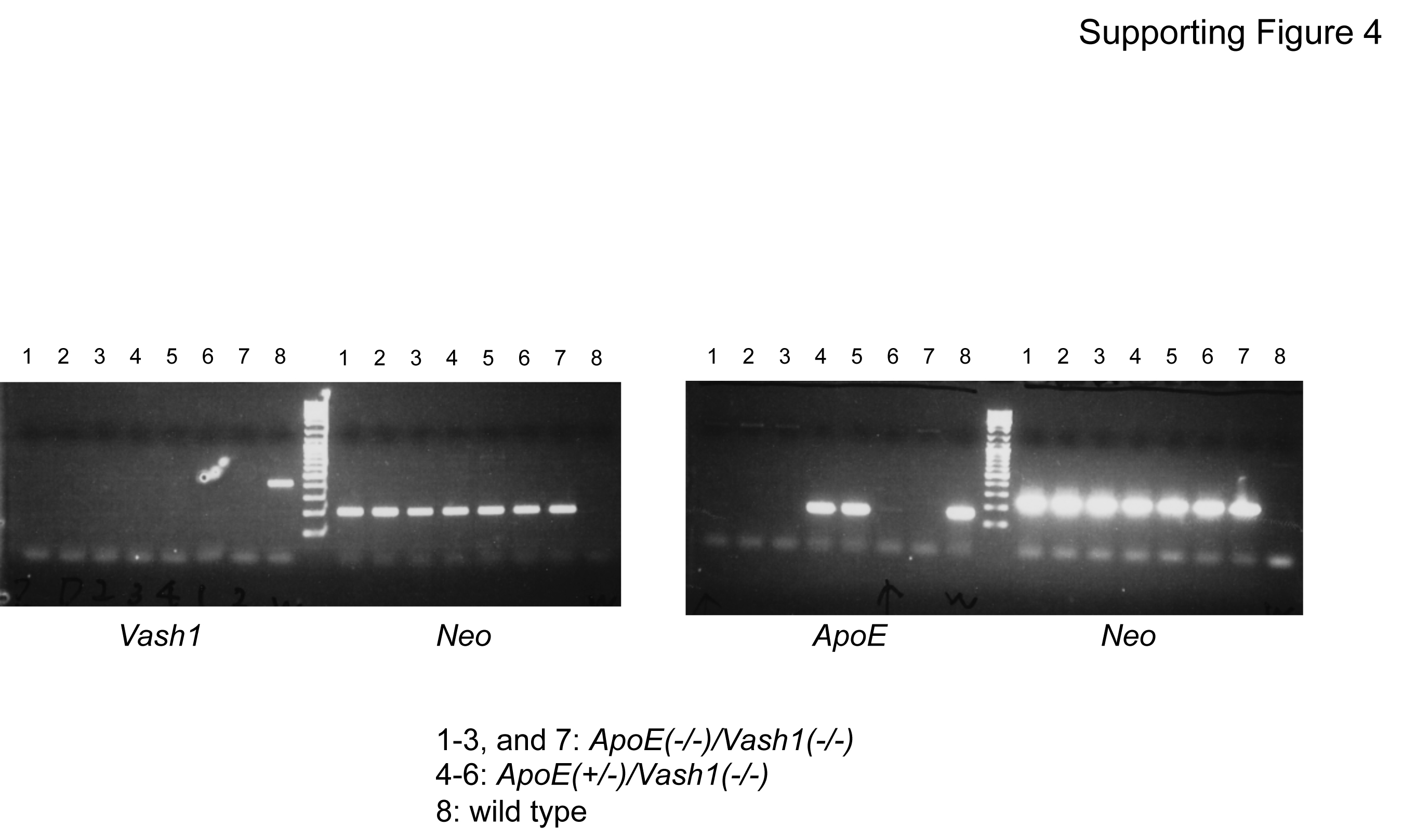

Supplement: Supplementary file 4 — Fig. S4 Genotyping of mice. [file ACEL-15-885-s004.jpg]
