# Supplementary material for: Age‐associated downregulation of vasohibin‐1 in vascular endothelial cells
Source: Aging Cell. 2016 Jun 21;15(5):885–92. doi: 10.1111/acel.12497 (PMC5013028; doi:10.1111/acel.12497)
Supplement: Supplementary file 5 — Table S1 Primer list. [file ACEL-15-885-s005.docx]

**Supporting Information**

Table S1. Primer list

| Genes | Forward（5’→3’） | Reverse（5’→3’） |
| --- | --- | --- |
| hVASH1 | AACTACTTCCGCCACATCGT | GGCGGCTTGTACATCAGGTC |
| mVASH1 | GATTCCCATACCAAGTGTGCC | ATGTGGCGGAAGTAGTTCCC |
| ICAM-1 | GTGATGCTCAGGTATCCATC | GTCCACTCTCGAGCTCATC |
| VCAM-1 | CAGGCTGGAGATTGATCTG | GAGAGATGTAGAGTTGTAGTTC |
| F4/80 | CATCATGGCATACCTGTTCAC | GAATGGGAGCTAAGGTCAGTC |
| MCP-1 | ACTGAAGCCAGCTCTCTCTTCCTC | TTCCTTCTTGGGGTCAGCACAGAC |
| PDGFβ | TGAAATGCTGAGCGACCA | GCTCGGGTCATGTTCAAGT |
| SDF-1α | GGTTCTTCGAGAGCCACAT | TTCGGGTCAATGCACACT |
| VEGFA | AGCACAGCAGATGTGAATGC | AATGCTTTCTCCGCTCTGAA |
| CD31 | TTCAGCGAGATCCTGAGGGTC | CGCTTGGGTGTCATTCACGAC |
| p16 | CACCGAATAGTTACGGTCGG | GCACGGGTCGGGTGAGAGTG |
| SIRT1 | TGGCAAAGGAGCAGATTAGTAGG | CTGCCACAAGAACTAGAGGATAAGA |
| β-actin | TCGTGCGTGACATCAAAGAG | TGGACAGTGAGGCCAGGATG |

Supporting Figure 1: Expression of p16 and SIRT1

Expressions of p16 and SIRT1 were compared between young and old HUVECs (N=3). Those expressions in old HUVECs are standardized as 1.0, and means±SDs are given. Experiments were performed twice.

Supporting Figure 2: Expression of various genes in the femoral arteries that might be related to neointimal formation

Samples were obtained from injured femoral arteries, and the expressions of the indicated genes were quantified and compared between WT and *VASH1^(-/-)^* mice: Inflammatory cytokines as well as M1 markers of macrophage (TNFα and MCP-1), M2 marker of macrophage (CD206), a factor related to VSMC proliferation (PDGF, adhesion molecules on ECs (ICAM-1 and VCAM-1), and stimulators of angiogenesis and macrophage mobilization (VEGF-A and SDF1. Means±SDs are shown (n=4~7).

Supporting Figure 3: Biochemical analysis of sera *ApoE^(-/-)^* and *ApoE^(-/-)^/Vash1^(-/-)^* mice

Total cholesterol (T-CHO), high-density lipoprotein cholesterol (HDL-C), triglyceride (TG), total lipid (TL), non-esterified fatty acid (NEFA), phospholipid (PL), aspartate aminotransferase (AST), alanine aminotransferase (ALT), lactate dehydrogenase (LDH), choline esterase (ChE), and blood glucose (Glu) were determined and compared. Means±SDs are shown (*ApoE^(-/-)^ mice*; N=16, *ApoE^(-/-)^/Vash1^(-/-)^*; N=6).

Supporting Figure 4: Genotyping of mice

Genotyping was examined by *Vash1, ApoE or Neo.* Number 8 was included as a control wild type mouse.
